# Nationwide Outbreak of *Candida auris* Infections Driven by COVID-19 Hospitalizations, Israel, 2021–2022^1^

**DOI:** 10.3201/eid2907.221888

**Published:** 2023-07

**Authors:** Roni Biran, Regev Cohen, Talya Finn, Tal Brosh-Nissimov, Galia Rahav, Dafna Yahav, Sharon Amit, Yael Shachor-Meyouhas, Alaa Atamna, Jihad Bishara, Liat Ashkenazi-Hoffnung, Haim Ben Zvi, Mirit Hershman-Sarafov, Shlomo Maayan, Yasmin Maor, Orna Schwartz, Oren Zimhony, Jonathan Lellouche, Meital Elbaz, Ela Burdelova, Naama Mizrahi, Anna Novikov, Oryan Henig, Ronen Ben-Ami

**Affiliations:** Tel Aviv Sourasky Medical Center, Tel Aviv, Israel (R. Biran, M. Elbaz, E. Burdelova, N. Mizrahi, A. Novikov, O. Henig, R. Ben-Ami);; Hillel Yaffe Medical Center, Hadera, Israel (R. Cohen);; Technion, Haifa, Israel (R. Cohen, Y. Shachor-Meyouhas, M. Hershman-Sarafov);; Sanz Medical Center, Netanya, Israel (T. Finn, J. Lellouche);; Samson Assuta Ashdod University Hospital, Ashdod, Israel (T. Brosh-Nissimov);; Ben Gurion University in the Negev, Beer Sheba, Israel (T. Brosh-Nissimov);; Sheba Medical Center, Tel Hashomer, Israel (G. Rahav, D. Yahav, S. Amit);; Tel Aviv University, Tel Aviv (G. Rahav, D. Yahav, A. Atamna, J. Bishara, L. Ashkenazi-Hoffnung, H. Ben Zvi, Y. Maor, R. Ben-Ami);; Rambam Medical Center, Haifa (Y. Shachor-Meyouhas);; Beilinson Hospital, Rabin Medical Center, Petach-Tikva, Israel (A. Atamna, J. Bishara);; Schneider Children’s Medical Center, Petach-Tikva (L. Ashkenazi-Hoffnung);; Bnai Zion Medical Center, Haifa (M. Hershman Sarafov); Barzilai Medical Center, Ashdod (S. Maayan);; Hebrew University of Jerusalem, Jerusalem, Israel (S. Maayan, O. Zimhony);; Wolfson Medical Center, Holon, Israel (Y. Maor, O. Schwartz);; Kaplan Medical Center, Rehovot, Israel (O. Zimhony); Ariel University, Ariel, Israel (J. Lellouche).

**Keywords:** *Candida auris*, COVID-19, coronavirus disease, SARS-CoV-2, severe acute respiratory syndrome coronavirus 2, viruses, respiratory infections, zoonoses, epidemiology, antifungal susceptibility, multilocus sequence typing, drug resistance, fungi, Israel

## Abstract

We report an outbreak of *Candida auris* across multiple healthcare facilities in Israel. For the period of May 2014–May 2022, a total of 209 patients with *C. auris* infection or colonization were identified. The *C. auris* incidence rate increased 30-fold in 2021 (p = 0.00015), corresponding in time with surges of COVID-19–related hospitalization. Multilocus sequence typing revealed hospital-level outbreaks with distinct clones. A clade III clone, imported into Israel in 2016, accounted for 48.8% of typed isolates after January 2021 and was more frequently resistant to fluconazole (100% vs. 63%; p = 0.00017) and voriconazole (74% vs. 5.2%; p<0.0001) than were non–clade III isolates. A total of 23% of patients had COVID-19, and 78% received mechanical ventilation. At the hospital level, outbreaks initially involved mechanically ventilated patients in specialized COVID-19 units and then spread sequentially to ventilated non–COVID-19 patients and nonventilated patients.

*Candida auris* is a drug-resistant fungal pathogen that has emerged over the past decade as a cause of nosocomial outbreaks with substantial mortality rates (*1*–*3*). Widespread resistance to triazole antifungals, rapid spread and persistence within hospital and nursing home environments, and difficulties in accurate identification by standard microbiological methods have prompted the US Centers for Disease Control and Prevention to list *C. auris* as a serious antibiotic-resistant threat (*4*). Recent reports of echinocandin-resistant and pan-resistant *C. auris* isolates in the United States and elsewhere have further heightened these concerns (*5*,*6*).

In 2014 and 2015, five patients with *C. auris* bloodstream infection were identified in 2 hospitals in the Tel Aviv, Israel, metropolitan area (*7*). After that outbreak, laboratory-based surveillance was initiated, and clinical isolates identified or suspected as *C. auris* were sent to the national mycology reference laboratory for sequence-based identification and antifungal drug susceptibility testing. Surveillance showed a stable low incidence of *C. auris* infections and no notable nosocomial clusters. An outbreak in 2016 that was limited to a single hospital originated in a patient transferred to Israel from a hospital in South Africa (*8*).

Since January 2021, a marked increase in the number of *C. auris* isolates referred to the national reference laboratory was noted; many medical centers reported *C. auris* infections for the first time. We conducted a nationwide survey of *C. auris* infections in Israel to assess clinical and microbiological characteristics and to determine drivers of epidemiologic change during 2021–2022.

## Methods

### Study Design and Population

After the first detection of *C. auris* in Israel in 2014, an alert was issued to all clinical microbiology laboratories to refer yeast isolates identified or suspected as *C. auris* to the national mycology reference laboratory at the Tel Aviv Sourasky Medical Center. Guidance on contact isolation, contact tracing, and environmental disinfection was provided to facilities that reported *C. auris* cases (*9*,*10*). 

This nationwide retrospective observational study covered the period January 1, 2014–May 31, 2022. We included all medical facilities that reported >1 *C. auris* clinical isolate during the study period. Yeast isolates sent to the reference laboratory underwent confirmatory DNA-sequence based identification, sequence typing, and antifungal susceptibility testing. Demographic and clinical data were collected from each site.

The study was reviewed and approved by the ethics committee of each participating medical center (approval no. for principal site 0543-21-TLV). Requirement for informed consent was waived because of the retrospective observational nature of the study.

### Data Collection

We extracted data from the hospital electronic medical records and laboratory computerized database by using a structured form. Collected data included demographics, comorbidities (quantified using the Charlson comorbidity score) (*11*), SARS-CoV-2 infection, previous exposure to antibacterial and antifungal drugs, infection with or carriage of drug-resistant organisms, and mechanical ventilation. Clinical outcomes were all-cause in-hospital death, length of hospitalization, length of stay in intensive care unit (ICU), and duration of mechanical ventilation. *C. auris* was considered a colonizer if growing from respiratory tract, skin, or rectal specimens and potentially clinically significant if isolated from normally sterile specimens.

### Microbiological Analyses

The general practice of microbiological laboratories is to identify yeasts cultured from normally sterile sites. Basic identification in participating laboratories was done by using CHROMagar *Candida* plates (CHROMagar, https://www.chromagar.com) and Vitek 2 with the YST ID card (bioMérieux, https://www.biomerieux.com). Hospital H1 implemented measures to identify *C. auris* carriers because of a large outbreak at that site. Those measures included identifying yeast isolates from all specimens and screening patients at admission to ICU and step-down units and at time of transfer from departments where *C. auris* cases were detected. *C. auris* screening was done by swabbing 3 sites (axilla, groin, and throat). Endotracheal aspirates were sampled from intubated patients. Swabs were inoculated onto Sabouraud dextrose agar and incubated at 37°C for 5 days.

*Candida* species identification at the reference laboratory was done using PCR and sequencing of the ribosomal DNA internal transcribed spacer (ITS) 1–5.8S-ITS2 and D1/D2 regions (*7*,*12*). Antifungal susceptibility testing was performed using broth microdilution according to Clinical and Laboratory Standards Institute guidelines (*13*). Antifungals tested were fluconazole, itraconazole, voriconazole, amphotericin B, and anidulafungin (obtained from Sigma, https://www.sigmaaldrich.com). Tentative *C. auris* susceptibility breakpoints for fluconazole (>32 mg/L), anidulafungin (>4 mg/L), and amphotericin B (>2 mg/L) were used, as proposed by the Centers for Disease Control and Prevention (*14*). Because no breakpoint has been defined for voriconazole, the epidemiologic cutoff (ECOFF) value was calculated from the MIC distribution using the ECOFF Finder program (*15*,*16*).

We determined genetic relatedness among *C. auris* isolates by using multilocus sequence typing, as described previously (*17*,*18*). We used Bayesian inference in MrBayes version 3.2 (*19*) to amplify, concatenate, and compare genomic sequences of RNA polymerase II (RPB)1, RPB2, internal transcribed spacer (ITS), and the D1/D2 region of the 28S nuclear ribosomal large subunit rRNA gene among strains. We used allele sequences reported by Kwon et al. (*17*) as references to classify strains into 4 main clusters (clades). We constructed minimum-spanning trees in R version 4.2.1 (R Foundation for Statistical Computing, https://www.r-project.org) and deposited all sequences into GenBank (Appendix 1).

### Statistical Analyses

We summarized patient characteristics by using descriptive statistics. We determined between-group differences by using the Fisher exact test for categorical variables and Student t or Wilcoxon rank-sum test for normally or nonnormally distributed continuous variables. We measured variance across multiple groups by using the Kruskal-Wallis test. The significance level (type I error) was set at 0.05. We performed all calculations in R.

## Results

**Figure 1:**
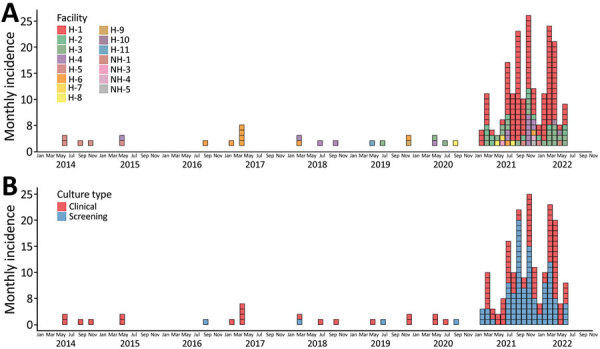
Incidence (no. cases) of *Candida auris* infection by medical facility (A) and type of specimen (B), Israel, 2014–2022. Epidemic plots were constructed with each patient appearing once, on the date of the first *C. auris*–positive specimen. H, hospital; NH, nursing home.

We recorded 209 patient-specific *C. auris* isolates during the 8-year study period. The first cases of *C. auris* infection in Israel were detected in May 2014 at a tertiary-level medical center in Tel Aviv. During May 2014–December 2020, a total of 24 cases of *C. auris* infection were reported from 7 hospitals (median incidence 4 cases/y, range 1–5 cases/y). The incidence of *C. auris* infection increased dramatically in 2021; an annual incidence of 120 cases was reported from 10 hospitals and 3 long-term care facilities, which represented a 30-fold increase over the previous base annual incidence (p = 0.00015; Figure 1). Of 185 patient-specific isolates identified beginning in January 2021, a total of 172 (92.9%) occurred in 4 community hospitals (H1–H4). Hospital H1, located in the Northern Central District of Israel, was the focus of the largest outbreak; 127 patient-specific *C. auris* isolates were reported (Figure 1, panel A). Repeat cultures were performed for 152 patients; colonization was detected for a median of 14 days (interquartile range [IQR] 4–32 days, maximum 210 days) (Appendix 2 Figure).

**Figure 2:**
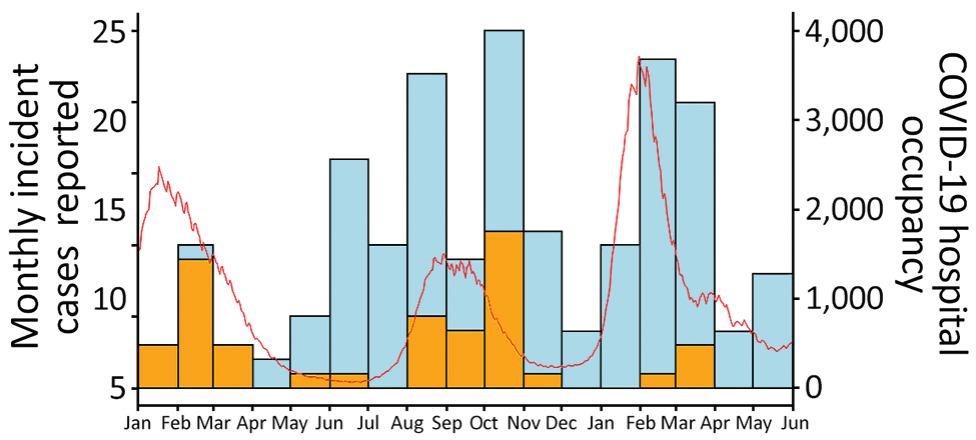
Association of *Candida auris* infection with COVID-19 hospitalization, Israel, January 2021–May 2022. Bars represent monthly *C. auris* incidence (no. cases). Cases with SARS-CoV-2 co-infection are shown in orange, non–co-infected cases are in blue. Red line shows level of hospital occupancy with COVID-19 patients. Scales for the y-axes differ substantially to underscore patterns but do not permit direct comparisons.

The incidence of *C. auris* cases during 2021 and 2022 corresponded with surges in COVID-19 cases in Israel during that period (Figure 2). *C. auris* cases peaked in January–March 2021, synchronous with the COVID-19 Alpha variant wave; in June–November 2021, matching the Delta variant wave; and in January–May 2022, during the Omicron wave. During the Alpha wave, 88.0% of patients with *C. auris* (15/17) were infected with SARS-CoV-2. That percentage decreased to 22% (23/103) during the Delta wave and 6.2% (4/65) during the Omicron wave (Figure 2).

### Strain Relatedness

**Figure 3:**
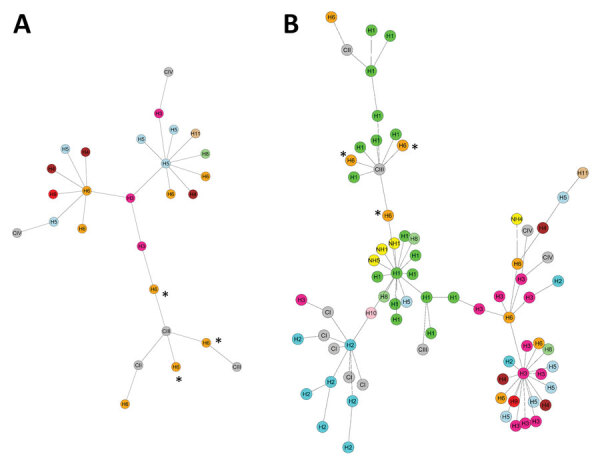
Minimum spanning trees of *Candida auris* strains for 2014–2020 (A) and 2014–2022 (B), Israel. Genetic relatedness of *C. auris* isolates was assessed using multilocus sequence typing. Strain cluster designation was determined using sequences published by Kwon et al. (shown in gray nodes) (*17*). Nodes are colored according to the respective medical center. Nodes marked with asterisks represent 2016 importation event from South Africa. C, clade; H, hospital; NH, nursing home.

**Figure 4:**
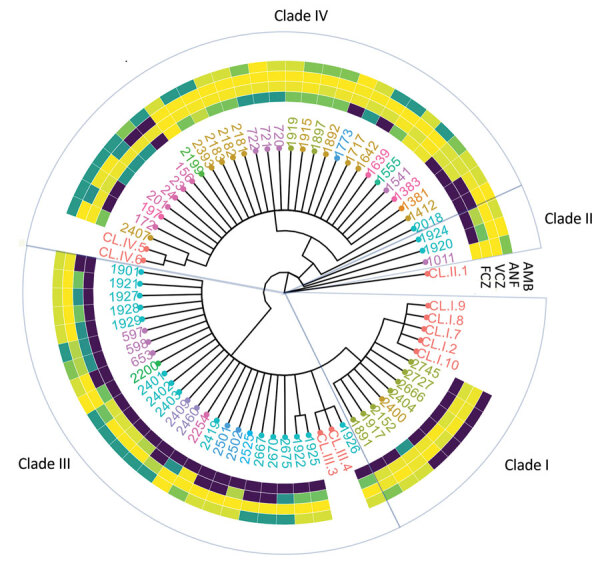
Association of population strain clustering with antifungal drug MIC and medical facility for *Candida auris* strains, Israel. A phylogenetic tree of *C. auris* isolates was constructed using multilocus sequence typing and Bayesian inference. Text colors represent different medical centers. Heat map colors represent MIC of each drug, ranging from fully susceptible (yellow) to resistant (dark blue). AMB, amphotericin B; ANF, anidulafungin; FCZ, fluconazole; VCZ, voriconazole.

We assessed the genetic relatedness and clade designation of *C. auris* strains isolated before 2021 and those from the 2021–2022 outbreak by using multilocus sequence typing. We typed 22 isolates collected during May 2014–December 2020; of those, 18 (81.8%) isolates were clade IV, 3 (13.6%) were clade III, and 1 (4.5%) was clade II (Figures 3, 4). The 3 clade III isolates represented an importation event in 2016 traced to a patient who acquired a surgical site infection in South Africa before being evacuated to Israel. The population structure changed after January 2021, when localized hospital outbreaks of clade III and clade I occurred. Of 43 isolates typed during January 2021–May 2022, a total of 24 (55.8%) belonged to clade III, 11 (25.5%) to clade IV, and 8 (18.6%) to clade I (Figures 3, 4). All isolates typed from hospital H1 (n = 18) belonged to clade III. Clade I isolates (n = 8) were identified in 2 hospitals (H2 and H3) in Israel’s Southern district. Clade III isolates from the 2016 outbreak clustered with the earliest 2021 H1 isolates, suggesting an epidemiologic link.

### Antifungal Susceptibility

**Figure 5:**
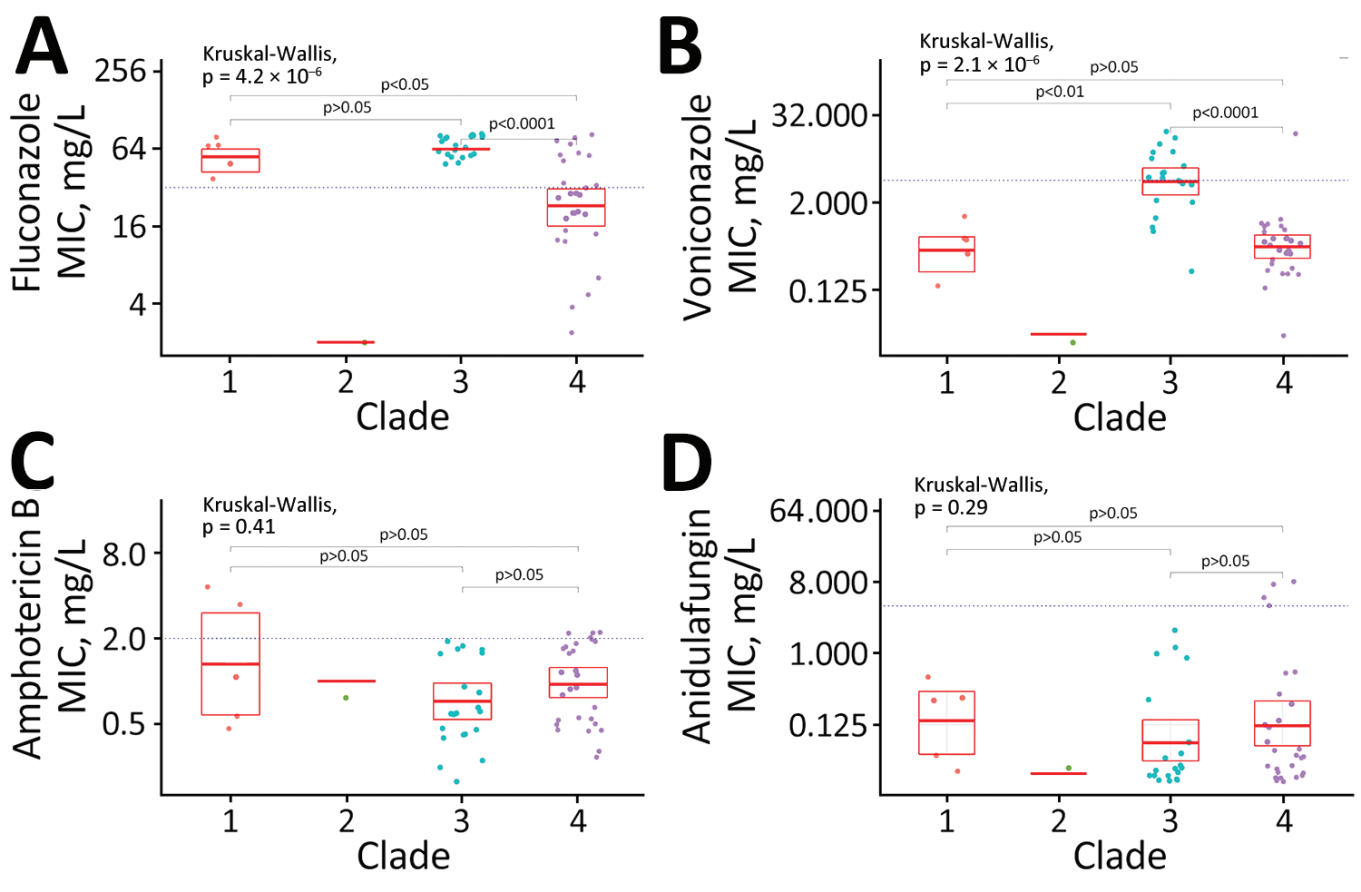
Comparison of antifungal MIC distribution among *Candida auris* clades, Israel. Antifungal MICs were determined using Clinical Laboratory Standards Institute M27A3/S4 methodology, for fluconazole (A), voriconazole (B), amphotericin B (C), and anidulafungin (D). Comparison among *C. auris* clades was done using the Kruskal-Wallis test and the pairwise Wilcoxon post-hoc test. Each dot represents a patient-specific isolate. Horizontal bars within box plots indicate medians, box tops and bottoms represent 95% confidence interval. NS, not significant.

**Figure 6:**
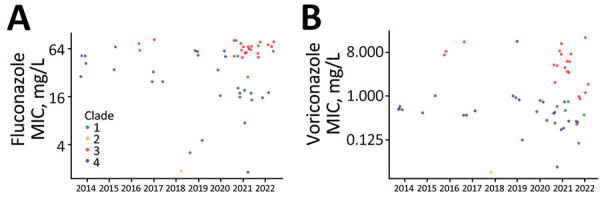
Temporal distribution of *Candida auris* clades and their respective MIC values, Israel. Distribution of *C. auris* fluconazole and voriconazole MIC is shown for January 2014–May 2022. Each dot represents a patient-specific isolate. High fluconazole and voriconazole MIC associated with clade III is noted in 2021 and 2022.

Overall rates of antifungal drug susceptibility were 15.5% (32/206) for fluconazole, 79.6% (164/206) for voriconazole, 86.4% (178/206) for amphotericin B, and 98.0% (198/202) for anidulafungin. A total of 21 isolates (10.0%) were resistant to both amphotericin B and fluconazole; 2 (0.95%) were resistant to fluconazole, amphotericin B, and anidulafungin. Clade III isolates had significantly higher MICs of fluconazole and voriconazole compared with non–clade III isolates (Figures 4,5,6). Resistance to fluconazole (MIC >32 mg/L) was detected in 100% (27/27) of clade III isolates versus 63.1% (24/38) of non–clade III isolates (p = 0.00017). Voriconazole MIC values above the calculated ECOFF (MIC >4 mg/L) were detected in 74.0% (20/27) of clade III isolates versus 5.2% (2/38) of non–clade III isolates (p<0.0001).

### Clinical Characteristics and Outcomes

Clinical data were available for 177 patients (86.7%) (Table). Patients were predominantly men (68.3%); median age was 70 years (IQR 55–80 years). Patients had multiple comorbidities; 50% had significant functional impairment and 30% had dementia. Most patients (78%) required mechanical ventilation during the same hospitalization, and 67% had a central venous catheter. Carriage or infection with other drug-resistant organisms was detected in 55% of patients.

**Table:** Clinical characteristics of patients infected or colonized with *Candida auris*, Israel

Characteristic	COVID-19	Non–COVID-19	p value	Total
No. patients	41 (23.2)	136 (76.8)		177 (100)
Median age, y (IQR)	67 (53–75)	72 (56–82)	0.16	70 (55–80)
Sex				
M	24 (58.5)	97 (71.3)	0.13	121 (68.3)
F	17 (41.4)	39 (28.6)		56 (31.6)
Long-term care facility	5 (12.1)	33 (24.2)	0.12	38 (21.5)
Functional status			0.0036	
Independent	30 (73.1)	59 (43.3)		89 (50.3)
Requires assistance	4 (9.7)	21 (15.4)		25 (14.1)
Complete dependence	7 (17.0)	56 (41.1)		63 (35.6)
Comorbidities				
Dementia	6 (14.6)	46 (33.8)	0.019	52 (29.4)
Malignancy	3 (7.3)	18 (13.2)	0.41	21 (11.8)
Median Charlson comorbidity score (IQR)	2 (0–3)	3 (1–4)	0.00016	2 (1–4)
Drug-resistant organism carriage/infection				
Any	21 (51.2)	76 (55.8)	0.72	97 (54.8)
Vancomycin-resistant *Enterococcus*	5 (12.1)	17 (12.5)	1.0	22 (12.4)
Methicillin-resistant *Staphylococcus aureus*	10 (24.3)	25 (18.3)	0.38	35 (19.8)
Carbapenem-resistant Enterobacteriaceae	8 (19.5)	31 (22.7)	0.83	39 (22.0)
* Clostridioides difficile*	0	6 (4.4)	0.33	6 (3.4)
Exposure to antimicrobials				
Antibacterial	37 (90.2)	119 (87.5)	0.78	156 (88.1)
Azole	5 (12.1)	11 (8.0)	0.53	16 (9.0)
Echinocandin	2 (4.8)	3 (2.2)	0.32	5 (2.8)
Amphotericin B	0	0	1.0	0
COVID-19 severity and treatment				
Critical	30 (73.1)	NA		30 (76.9)
Severe	8 (19.5)	NA		8 (20.5)
Mild	1 (2.4)	NA		1 (2.5)
Corticosteroids	38 (97.4)	NA		38 (97.4)
Remdesivir	17 (43.5)	NA		17 (43.5)
Treatment required				
Intensive care unit	14 (34.1)	34 (25.0)	0.31	48 (27.1)
Mechanical ventilation	32 (78.0)	106 (77.9)	1.0	138 (78.0)
Central venous catheter	30 (73.1)	89 (65.4)	0.44	119 (67.2)
Outcome				
Median hospital stay, d (IQR)	36 (24–52)	36 (21–54)	0.98	36 (21.5–54)
Median ICU stay, d (IQR)	24 (18.5–38)	21 (11.2–28.8)	0.08	21.5 (13–33)
Median mechanical ventilation duration, d (IQR)	35.5 (19.8–46)	35 (17.8–51.2)	0.60	35 (18–50)
In-hospital death	15 (36.5)	55 (40.4)	0.71	70 (40.0)

*Values are no. (% within COVID-19 group) except as indicated. IQR, interquartile range; NA, not applicable.

Forty-one patients (23.2%) had received a COVID-19 diagnosis before acquiring *C. auris* in the same hospital stay. Most of those patients (73.1%) had critical COVID-19. Almost all patients with COVID-19 received corticosteroids, and half were treated with remdesivir. The median time from detection of SARS-CoV-2 infection to recovery of *C. auris* was 25 days (IQR 11–38.5 days). Patients with and without COVID-19 had similarly high rates of mechanical ventilation (78%), but patients with COVID-19 had better baseline functional status, fewer comorbidities, and lower rates of dementia (Table).

Of 177 patients, 82 (46.3%) had positive clinical specimens, and 95 (53.6%) were colonized with *C. auris* with no evidence of invasive candidiasis. The proportion of colonized versus infected patients was significantly greater for patients with COVID-19 (70.7% vs. 48%; p = 0.013) and in hospital H1, where screening was implemented (77.7% vs. 14.9% in other hospitals; p<0.0001). Clinical specimens consisted of urine (59.8%, n = 49), blood (36.6%, n = 30), and wounds (17.1%, n = 14). In-hospital death occurred in 70 (39.5%) patients. The in-hospital mortality rate did not differ significantly between patients with clinical infections, including those with *C. auris* bloodstream infections, and patients who were only colonized with *C. auris*. Increasing age and comorbidity (Charlson score) were predictors of in-hospital death (Appendix 2). Of the surviving patients, 27 (29.0%) were discharged to home, 27 (29.0%) to ventilator-capable skilled nursing facilities, 19 (20.4%) to rehabilitation facilities, and 17 (18.2%) to long-term care facilities.

### Time Course of *C. auris* Hospital Outbreaks

**Figure 7:**
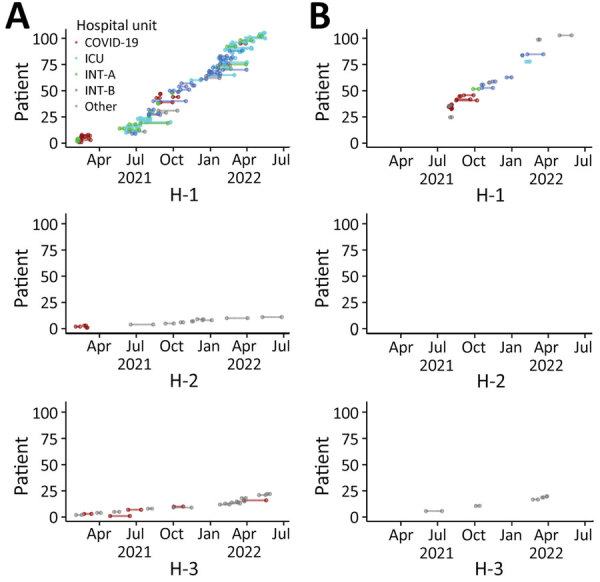
Hospital-level outbreak time course for the 3 hospitals with the largest *Candida auris* case numbers, Israel. Time course of *C. auris* hospital outbreaks is shown for patients receiving mechanical ventilation (A) and patients not receiving mechanical ventilation (B). Individual patients are displayed in bottom to top order according to the first date they became infected. Bars are plotted from the date of first *C. auris*–positive specimen to the date of hospital discharge. Colors represent hospital unit at the time *C. auris* was recovered. COVID-19, specialized COVID-19 unit; H, hospital; ICU, intensive care unit; INT-A, intermediate care unit A; INT-B, intermediate care unit B.

We analyzed the evolution of the *C. auris* outbreaks in hospitals H1, H2, and H3 (Figure 7). In H1, *C. auris* infections were first detected among 10 mechanically ventilated COVID-19 patients; 9 of these infections occurred over a period of 13 days in February 2021 (cluster 1). Next, *C. auris* infections were detected in mechanically ventilated patients with no history of COVID-19 (cluster 2). Infections were first detected in May 2021 in intermediate care unit A, to which convalescing COVID-19 patients had been transferred, and in the adjacent general ICU. The first of the non–COVID-19 cases was a patient admitted 52 days after the last of the COVID-19 patients had been discharged. Additional cases were detected in intermediate care unit B starting in July 2021, after that unit became a destination for recovering COVID-19 patients. Overall, 65 mechanically ventilated patients were infected with *C. auris* in cluster 2.

A similar pattern, in which a cluster of *C. auris* cases in mechanically ventilated patients with COVID-19 was followed by spread to ventilated patients without COVID-19, was observed in hospital H2, albeit on a smaller scale. The gap between discharge of the last cluster 1 patients and admission of the first cluster 2 patient was 67 days. Cluster 1 strains included 2 sequence types belonging to clade I and clade IV, whereas only the clade I sequence type was identified in cluster 2. In hospital H3, *C. auris* infection was detected predominantly in ventilated patients, with or without COVID-19, with no temporal clustering over the study period (Figure 7).

## Discussion

We report an ongoing nationwide outbreak of *C. auris* colonization and infection in hospitals in Israel. The introduction of distinct clones of clade I and clade III into 3 hospitals, as well as increased circulation of clade IV, resulted in a 30-fold increase in the annual *C. auris* incidence rate in 2021. Neither clade I nor clade III had circulated in Israel before 2021, suggesting they arose through importation events into the country. Further, phylogenetic analyses showed that the clade III isolates collected during the current outbreak were related to those imported into Israel from South Africa in 2016 (*8*). The shift in clade distribution was associated with a change in the azole MIC range; specifically, clade III strains had higher fluconazole and voriconazole MICs compared with those for clade IV strains. Thus, the continuing expansion of clade III in Israel and its spread beyond H1 to other medical facilities might limit the already constrained treatment options for *C. auris*.

We identified 2 main drivers of *C. auris* healthcare-associated dissemination in this outbreak. The first was COVID-19. Almost one quarter of patients with *C. auris* infection or colonization were infected with SARS-CoV-2 and received care in designated COVID-19 units. *C. auris* incidence rates corresponded in time with COVID-19–related surges in hospitalization. Cases of *C. auris* infection in COVID-19 wards tended to be tightly clustered (Figure 7), suggesting efficient healthcare-associated transmission within those units. Multiple genotypes of *C. auris* were found in COVID-19 units in hospitals H1 and H2, and 1 dominant clone carried over to non–COVID-19 patients in other departments. Outbreaks of *C. auris* have been reported in COVID-19 care units in the United States, India, Mexico, and Columbia, resulting in colonization or infection rates as high as 50% (*20*–*24*). Potential reasons for the susceptibility of COVID-19 units to such outbreaks include the use of double gloving (wearing two pairs of gloves), poor adherence to hand hygiene, and inadequate disinfection of shared medical devices and equipment (*20*).

A second crucial driver appeared to be mechanical ventilation. Patients with and without COVID-19 had similarly high rates of mechanical ventilation (78%). Moreover, within specific hospitals, *C. auris* spread first among mechanically ventilated COVID-19 patients and then infected non–COVID-19 patients in intermediate care units shared by both recovered COVID-19 and non–COVID-19 patients. This second population included patients with multiple comorbidities, high rates of dementia, and long-term mechanical ventilation. *C. auris* moved between those patient populations asynchronously; gaps of >6 weeks were observed in 2 hospitals between discharge of the last infected COVID-19 patient and admission of a ventilated non–COVID-19 patient who later acquired *C. auris*. Possible explanations include persistence of *C. auris* in the patient environment and on shared medical equipment, as well as undetected carriage by colonized patients or healthcare workers. *C. auris* was previously isolated from 70% of environmental samples at a ventilator-capable skilled nursing facility, including those from handrails, doorknobs, and windowsills (*25*). In vitro studies found that *C. auris* forms biofilm on plastic surfaces and is able to persist in viable colonies for >2 weeks and as viable nonculturable cells for >4 weeks (*26*).

Among the study cohort, 46% had clinical *C. auris* infection, including 30 patients with candidemia. The in-hospital mortality rate was 40% and was similar for patients colonized and infected with *C. auris*, likely reflecting the multiple acute and chronic comorbidities in this patient population. Of the surviving patients, only 30% were discharged home; the rest were transferred to ventilator-capable skilled nursing facilities, rehabilitation facilities, and long-term care facilities, potentially establishing reservoirs of *C. auris* carriage.

Limitations of this study include lack of systematic active surveillance and environmental sampling in most medical centers. In addition, hospitals differed in some key areas, including criteria for performing yeast species identification and screening for *C. auris* colonization.

In summary, *C. auris* is spreading in multiple hospitals in Israel, and appears set to become endemic in some facilities. The emergence and amplification of new *C. auris* clones was traced to patients receiving mechanical ventilation in COVID-19 isolation units. *C. auris* was transmitted from this population to non–COVID-19 patients in shared intermediate care units and from there disseminated to nonventilated patient populations. New guidelines addressing this public health threat were recently published by the Israeli Ministry of Health (*27*). Continued surveillance and implementation of infection control measures, focusing on debilitated patients and those receiving mechanical respiratory support, are essential to control the spread of *C. auris*.

Appendix 1 Additional data used in study of nationwide outbreak of *Candida auris* infections driven by COVID-19 hospitalizations, Israel, 2021–2022.

Appendix 2 Additional information about nationwide outbreak of *Candida auris* infections driven by COVID-19 hospitalizations, Israel, 2021–2022.
