# Supplementary material for: Nationwide Outbreak of Candida auris Infections Driven by COVID-19 Hospitalizations, Israel, 2021–2022
Source: Emerg Infect Dis. 2023 Jul;29(7):1302–11. doi: 10.3201/eid2907.221888 (PMC10310389; doi:10.3201/eid2907.221888)
Supplement: Appendix 2 — Additional information about nationwide outbreak of Candida auris infections driven by COVID-19 hospitalizations, Israel, 2021–2022. [file 22-1888-Techapp-s2.pdf]

Article DOI: https://doi.org/10.3201/eid2907.221888

EID cannot ensure accessibility for supplementary materials supplied by authors. Readers who have difficulty accessing supplementary content should contact the authors for assistance.

Nationwide Outbreak of *Candida auris* Infections Driven by COVID-19 Hospitalizations, Israel, 2021–2022

Appendix 2

Appendix 2 Table 1. Variables associated with in-hospital mortality

Category	Dead N = 70 (40.0)	Alive N = 105 (60.0)	P
Age, years, median (IQR)	76 (64–83)	68 (50–78)	0.0084
Sex			0.25
Female	26 (46.4)	30 (53.6)	
Male	44 (36.4)	75 (62.0)	
Charlson score, median (IQR)	3 (1–5)	2 (1–3)	0.042
Dementia	19 (36.5)	31 (59.6)	0.86
Functional impairment	35 (39.8)	51 (58.0)	0.87
C. auris			
Clinical infection	35 (42.7)	45 (54.9)	0.35
Bloodstream infection	15 (50)	15 (50)	0.22
Antifungal treatment	18 (50)	18 (50)	0.18
Echinocandin	13 (48.1)	14 (51.9)	0.39
Amphotericin B	4 (40)	6 (60)	1.0
Azole	9 (50)	9 (50)	0.44
COVID19	15 (36.5)	26 (63.4)	0.71
Mechanical ventilation	57 (41.3)	81 (58.7)	0.57

*Values represent numbers (percent) unless specified otherwise. IQR, interquartile range.

Appendix 2 Table 2. Variables associated with dementia

Category	Dementia	Control	P
N =	52 (29.3)	125 (70.6)	
C. auris			
Clinical infection	22 (42.3)	60 (48.0)	0.51
Colonization	30 (57.7)	65 (52.0)	
Bloodstream infection	10 (19.2)	20 (16.0)	0.66
COVID	6 (11.5)	35 (28)	0.019
Severe/critical	6 (11.5)	34 (27.2)	
Long-term mechanical ventilation	10 (19.2)	3 (2.4)	0.00032
In-hospital mortality	19 (36.5)	51 (40.8)	0.86

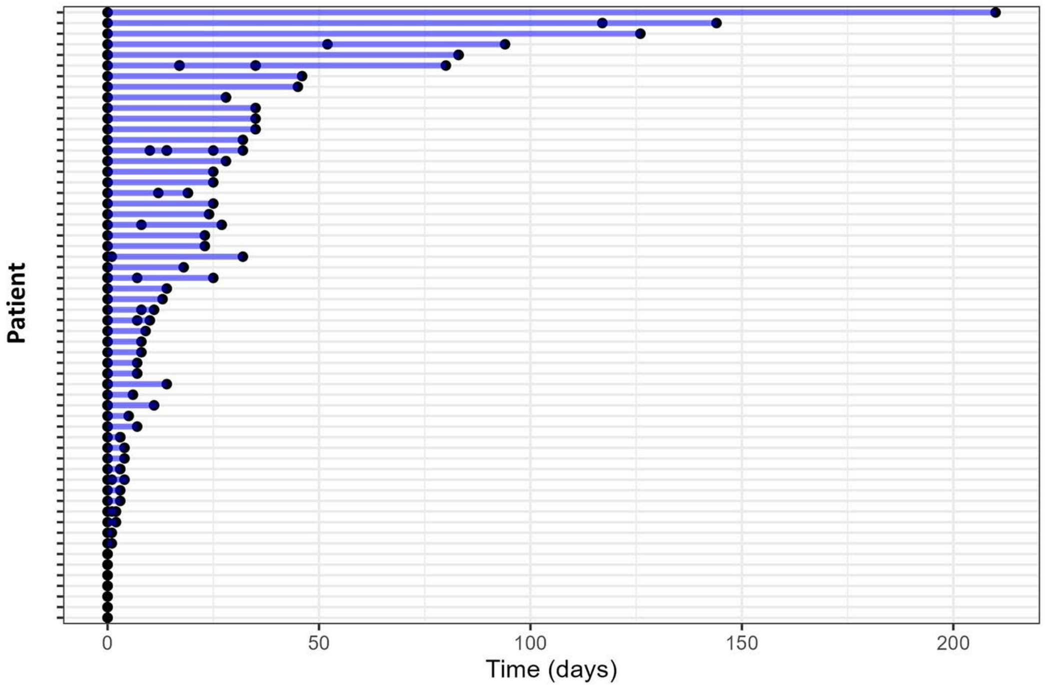

Appendix 2 Figure. Temporal distribution of positive *Candida auris* cultures in patients with repeat samples.
